# Wireless MRI Colonoscopy for Sensitive Imaging of Vascular Walls

**DOI:** 10.1038/s41598-017-03902-7

**Published:** 2017-06-26

**Authors:** Xianchun Zeng, Liangliang Chen, Chuan Wang, Jian Wang, Chunqi Qian

**Affiliations:** 1 Department of Radiology, Southwest Hospital, Third Military Medical University, Chongqing, China; 2 Department of Radiology, Guizhou Provincial People’s Hospital, Guiyang, China; 3 Department of Electrical and Computer Engineering, Michigan State University, East Lansing, MI USA; 4 Department of Radiology, Michigan State University, East Lansing, MI USA

## Abstract

A Wireless Amplified NMR Detector (WAND) with cylindrical symmetry has been fabricated and non-surgically inserted into a rodent lower digestive track to improve the imaging quality of deep-lying vessels inside the abdominal cavity. This symmetric detector has a compact design using two end-rings and two vertical legs to create two orthogonal resonance modes. Based on the principle of parametric amplification, the detector can harvest wireless pumping power with its end-rings and amplify Magnetic Resonance signals induced on its vertical legs. With good longitudinal and azimuthal homogeneity, the WAND can achieve up to 21-times sensitivity gain over a standard external detector for immediately adjacent regions, and at least 5-times sensitivity gain for regions separated by one diameter away from the detector’s cylindrical surface. The WAND can approach the region of interest through the lower digestive track, similar as a colonoscopy detector. But unlike an optical camera, the amplified MR detector can “see” across intestinal boundaries and clearly identify the walls of bifurcated vessels that are susceptible to atherosclerotic lesions. In addition to vascular wall imaging, this detector may also be used as a swallowable capsule to enhance the detection sensitivity of deep-lying organs near the digestive track.

## Introduction

Magnetic Resonance Imaging (MRI) can diagnose soft tissues lesions based on subtle changes of signal intensity in their pathological states. Such capability, however, is often hindered by the limited detection sensitivity, especially for regions deep lying inside the body. With the advent of higher magnetic fields^1^, array coils^2, 3^, faster imaging sequences^4, 5^ and hyperpolarization schemes^6, 7^, the detection sensitivity of MRI has continuously increased over the past few decades. Recently, a complementary strategy has been proposed to further enhance MRI detection sensitivity with a Wireless Amplified NMR Detector (WAND)^8–11^. This approach is based on the general concept that weak MR signals from a local region of interest can be more sensitively detected by a localized coil. But unlike traditional microcoils^12^ that normally require wired connections^13^ for signal transmission, the WAND has a built-in parametric amplifier^14–17^ that can harvest wirelessly provided pumping power to amplify MR signals *in situ*. Instead of relying on passive coupling^18–20^ where the signal transmission efficiency could rapidly decay over larger distance separations, *in situ* amplification can greatly improve the signal transmission efficiency over larger distance separations and enhance the local detection sensitivity^8^. The WAND was initially developed as an implantable detector to identify individual nephrons *in vivo* ^9, 10^. It was later used inside the esophagus to sensitively observe vascular walls in the neck and chest regions^11^. These previous versions of detectors had suboptimal homogeneity. They were unable to get panoramic views of their surroundings due to the lack of cylindrical symmetry. As a result, orientation adjustment was required to achieve reasonable homogeneity over a specified region. In this work, a new detector design with cylindrical symmetry is introduced to improve detection homogeneity. This symmetric detector can be non-surgically inserted from the rectal to sensitively image vascular walls that are deep lying inside the abdominal cavity, without the need for orientation adjustment. Compared to a standard external coil, the WAND can enhance detection sensitivity by up to 21-times for regions immediately adjacent to the detector’s surface. For regions that are farther away, the detector has at least 5 times sensitivity gain even when the distance separation between the region of interest and the detector’s cylindrical surface is large than its own diameter. Such detection capability enables high resolution imaging of bifurcated vessels with improved quality, paving way for longitudinal studies of subtle lesions on vascular walls. This detector can also be mounted on endoscopic catheters to sensitively diagnose tumor metastasis around the esophagus or colon. Moreover, this wireless detector could potentially be used as swallowable pill that can travel inside tortuous small intestines to “see” across intestinal walls at real time.

## Methods

### Detector Construction

**Figure 1:**
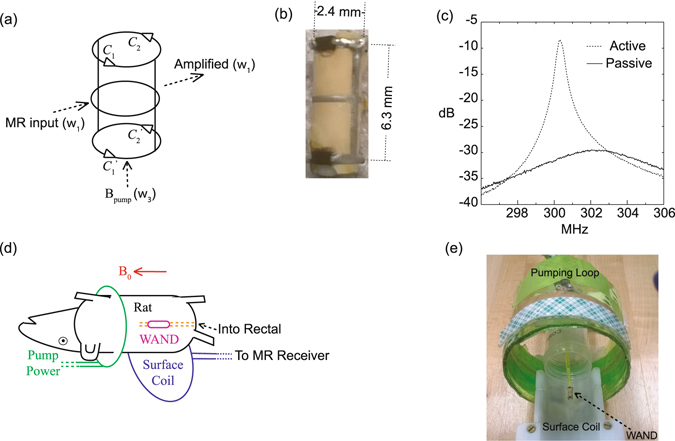
The operation of wireless powered amplification. (**a**) The schematic diagram of a cylindrically symmetric Wireless Amplified NMR Detector (WAND) with a horizontal (lower frequency) resonance mode and a longitudinal (high frequency) resonance mode. (**b**) The picture of a 6.3-mm long detector made of a patterned layer of copper clad polyimide wrapped around a 2.4-mm diameter polyurethane cylinder. (**c**) The solid curve is the *S*_21_ curve measured for the passive detector without pumping power, and the dotted curve is that measured for the active detector in the presence of pumping power at about 0.3 dB below the detector’s oscillation threshold. (**d**) A WAND device inserted inside the rat rectal with inductive coupling to the pumping loop and the external surface coil. (**e**) The arrangement for phantom test with the WAND inserted in the center of a water tube placed above the surface coil. The distance separation between the WAND and the external surface coil is about 22 mm.

The WAND can utilize circuit nonlinearity to directly convert wirelessly harvested energy into amplified MR signals^8^. The detector is normally a nonlinear double frequency resonator^9^ that can receive both the MR signal at the Larmor frequency *ω*_1_ and the pumping signal at approximately twice the Larmor frequency, i.e. *ω*_3_ ~ 2*ω*_1_. It is necessary to make |*ω*_3 _− *2ω*_1_| slightly larger than the imaging bandwidth, so that the amplified MR signal at *ω*_1_ doesn’t interfere with the “idler signal” at the difference frequency *ω*_2_ = *ω*_3 _− *ω*_1_. Previous versions of WANDs^8, 9, 11^ didn’t have very good cylindrical symmetry, because non-identical inductors were used to create multiple resonance modes. Here, a cylindrically symmetric detector is constructed out of two identical legs, two identical split end-rings and one continuous center ring (Fig. 1a). The two vertical legs create the lower-frequency mode that is sensitive to spin precession in the transverse plane. The two end-rings have their gaps bridged by four identical varactors to create the higher-frequency mode that is sensitive to a pumping field applied longitudinally. For each end-ring, the cathode of *C*_1_ is connected to the anode of *C*_2_, so that both varactors are modulated in the same direction by the pumping field. The continuous center ring connects the virtual grounds of both vertical legs, in order to neutralize accumulated charges without affecting the detector’s resonance frequencies. As shown in Fig. 1b, the WAND has a length of 6.3 mm. It is made of a patterned layer of copper clad polyimide wrapped around a 2.4-mm diameter polyurethane cylinder. The copper pattern has a strip width of 0.3 mm, whose end-rings are bridged by identical varactors (BAS3005A, Infineon). The *S*_21_ curve was measured with a double pick-up loop placed above the detector and connected to a network analyzer. The resonator’s quality factor *Q* was subsequently obtained by dividing the center frequency *f*_0_ of a resonance peak with its −3 dB bandwidth Δ*f*_−3dB_^21^. According to the *S*_21_ curve, the passive resonator has a higher resonance frequency at 602.0 MHz (*Q* = 30), which is approximately twice its lower resonance frequency at 302.3 MHz (*Q* = 50). When the resonator is activated by a longitudinal pumping field, the resonator’s *S*_21_ curve will have increased height and decreased band width. Equivalently, the effective quality factor *Q*_*eff*_ (defined as *f*_0_/ Δ*f*_−3dB_) will increase with increased pumping field. *Q*_*eff*_ will approach infinity when the wirelessly harvested pumping power completely compensates for circuit loss^8^. To have an amplifier with finite gain and finite bandwidth, the pumping power should be slightly reduced, i.e. at about 0.3 dB below the resonator’s oscillation threshold. At this time, the WAND has about 23-dB gain at the Larmor frequency at 300.3 MHz and a −3 dB bandwidth of ~0.4 MHz (Fig. 1c). This reduced bandwidth is still much larger than the imaging bandwidth used in most MRI experiments. For sensitivity comparison, a passive resonator optimized for 300 MHz operation was built with the same dimension as the WAND.

### MRI Experiments

The cylindrically symmetric detector was coated by a thin layer of medical grade epoxy and inserted into the detection object for *in situ* signal amplification. A four-element rat brain array (Bruker Biospin) was placed externally beneath the detection object to receive amplified signals. A single-turn pumping loop was aligned along the static magnetic field B_0_ (Fig. 1d), while the entire assembly (Fig. 1e) was inserted inside a 7 T horizontal-bore MRI scanner (Bruker Biospin). During RF excitation, the WAND was decoupled by the excitation pulse that can strongly modulate the capacitance of its constituting varactors. During signal acquisition, the pumping power was turned on to activate the resonator and improve the transmission efficiency. Normally, only ~10 mW of pumping power was required on the pumping coil to set the WAND approximately 0.3 dB below its oscillation threshold. This tiny amount of power would introduce negligible heating in the sample.

To evaluate the WAND’s performance in a water phantom, multi-slice 2D gradient echo images were acquired with the optimized passive resonator and the actively amplified resonator, using the following acquisition parameters: TR/TE = 250/3.5 ms, 30° flip angle, 28.8 × 28.8 mm^2^ FOV, 0.4 mm slice thickness, 288 × 288 matrix, 25 kHz imaging bandwidth. These images were compared to those images acquired with only the external surface coil but using the same parameter set. Subsequently, the double angle method^22^ was used to evaluate the WAND’s perturbation on excitation pulse homogeneity, using 60° and 120° excitations, TR = 5 s and all other parameters remaining the same.

During *in vivo* experiments, a 300-grams rat was anesthetized with isoflurane and secured in the supine position before the WAND was inserted into its rectal. The detector was connected to an insertion rod, whose insertion depth could be conveniently adjusted to bring the detector closer to the aortic bifurcation. To locate the relative position of the aortic bifurcation with respect to detector, transverse gradient-echo images were acquired using TR/TE = 58/2.5 ms, 35° flip angle, 4 × 4 cm^2^ FOV, 1 mm slice thickness, 256 × 256 matrix, 25 kHz bandwidth, NA = 1. Subsequently, longitudinal spin-echo images were acquired to identify vascular walls of the left and right common iliac arteries in the absence and presence of pumping power. Acquisition parameters for these longitudinal images were TR/TE = 1000/7.7 ms, 90° flip angle, 19.2 × 19.2 mm^2^ FOV, 1 mm slice thickness, 192 × 192 matrix, 50 kHz bandwidth, NA = 4.

To observe the Superior Mesenteric Artery (SMA) and the Celiac Artery (CA), the detector was further pushed inside until it appeared in the same transverse slice as the SMA. This transverse slice was acquired with gradient echo sequence using the following parameters: TR/TE = 153/2.9 ms, 35° flip angle, 25.6 × 25.6 mm^2^ FOV, 1 mm slice thickness, 256 × 256 matrix, 25 kHz bandwidth, NA = 1. Subsequently, longitudinal spin-echo images were acquired along a plane that simultaneously passed through the aorta, the SMA and the CA. Acquisition parameters for these longitudinal images were TR/TE = 1000/8.1 ms, 90° flip angle, 3 × 3 cm^2^ FOV, 1 mm slice thickness, 300 × 300 matrix, 50 kHz bandwidth, NA = 2.

All animal experiments were performed in accordance with guidelines set forth by the Institutional Animal Care and Use Committee (IACUC) at MSU. All experimental protocols were approved by the IACUC at MSU.

### Image Processing with MATLAB

To demonstrate the sensitivity advantage of the WAND in phantom studies, relative sensitivity maps were obtained by dividing sensitivity-normalized images acquired by the resonator with those acquired by the external surface coil. To delineate vascular walls for *in vivo* images, Canny’s method^23^ for edge detection was utilized to identify boundary regions with abrupt changes in signal intensity. After the magnitude gradient for each pixel in the sensitivity normalized image was calculated, a boundary pixel could be identified with 95% confidence level if the pixel’s magnitude gradient exceeds twice the standard deviation evaluated over the entire gradient plot.

### Data Availability

The datasets analyzed during the current study are available from the corresponding author upon request.

## Results

### Phantom Tests

**Figure 2:**
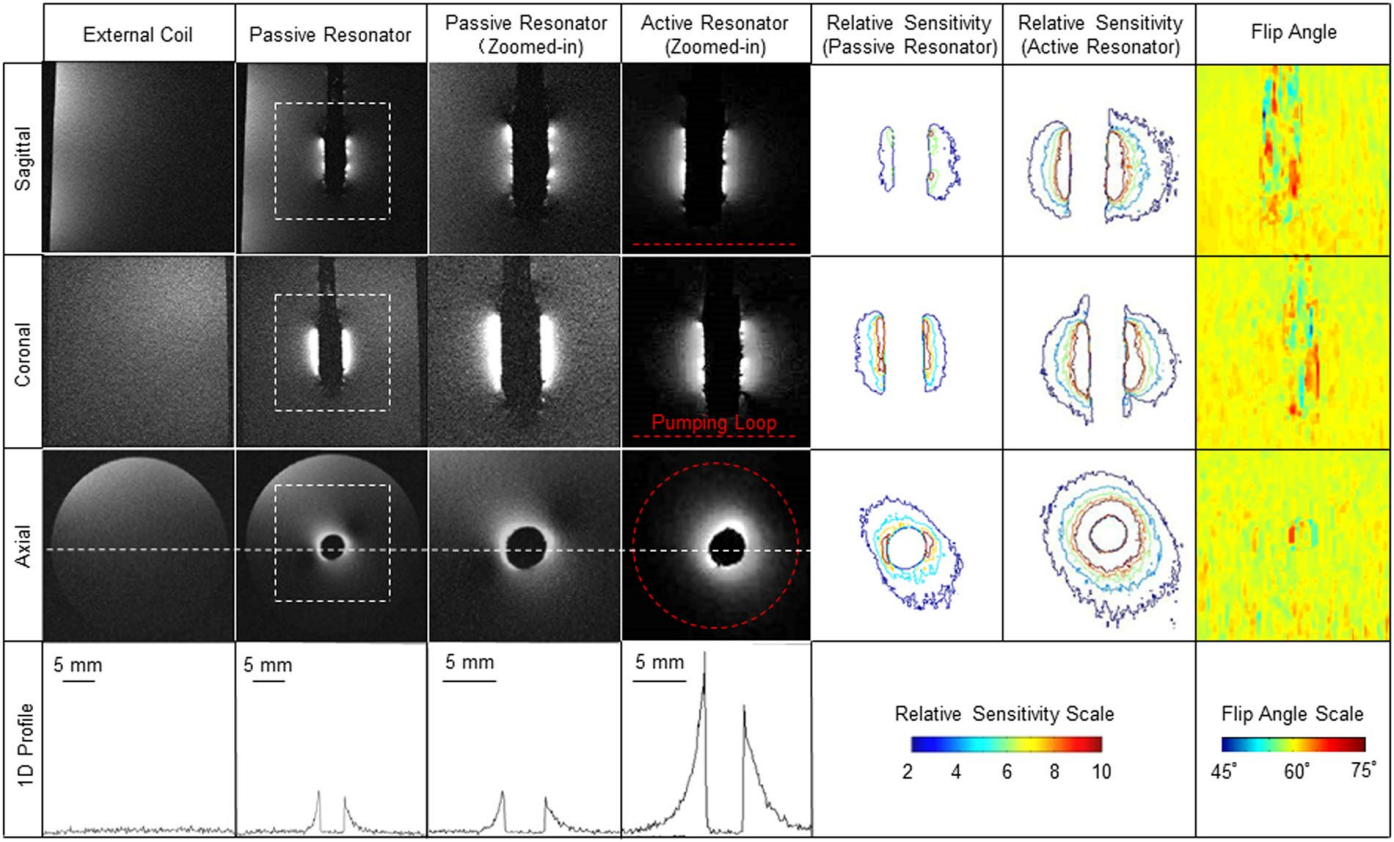
2D GRE images of a water phantom. Images in column 1 were acquired with the external surface coil only. Images in column 2 were acquired with the optimized passive resonator inserted. Column 3 shows zoomed-in views of image regions defined by the dashed square in column 2. Column 4 shows zoomed-in views acquired by the active resonator, with the pumping loop schematically represented by red dashed lines. Column 5 shows the relative sensitivity maps of images acquired by the optimized passive resonator with respect to those acquired by the external surface coil. Column 6 shows the relative sensitivity maps of images acquired by the active resonator with respect to those acquired by the external surface coil. Column 7 shows the zoomed-in view of flip angle maps around the resonator obtained by the double angle method^22^. The fourth row shows 1D SNR profiles taken along the dashed lines shown in the axial images in the third row.

Row 1, row 2 and row 3 in Fig. 2 show the sagittal, coronal and axial images acquired with the external surface coil (column 1), with the optimized passive resonator (column 2) and with the active resonator (column 3) respectively. According to the 1D intensity profiles in row 4, the passive resonator has up to 5-times the sensitivity of the external surface coil for regions immediately adjacent to the detector’s surface, owing to concentrated magnetic fluxes near the passive resonator^18, 24^. On the other hand, the active resonator has up to 21-times sensitivity gain for the same regions, primarily due to the improved signal transmission efficiency brought by *in situ* amplification. For regions farther away from the detector’s surface, the sensitivity advantage of the active resonator is more obvious. As shown by the relative sensitivity maps of the passive (column 5) and the active (column 6) resonator, the active resonator has a larger effective detection range with better azimuthal and longitudinal homogeneity. According to the transverse profile in column 6 row 3, the active resonator has at least 5 times sensitivity gain for a circular region up to 3 mm from the detector’s surface. This is a distance separation larger than the detector’s own diameter. Such a level of sensitivity gain is maintained over a 6-mm region in the longitudinal direction, as shown by the relative sensitivity maps in column 6 (rows 1 and 2). Moreover, the zoomed-in views of flip angle maps in column 7 show negligible perturbation of the detector on the uniformity of RF excitation, owing to the strong modulation of varactors by the excitation pulse.

### *In vivo* Images

**Figure 3:**
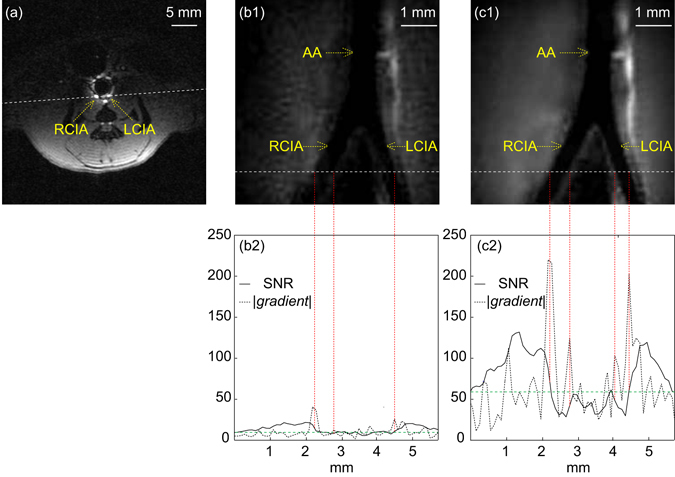
*In vivo* images of aortic bifurcation. (**a**) The transverse gradient-echo image to locate the aortic bifurcation with the passive resonator inserted inside the rectal. (b1) and (c1) are zoomed-in views of the longitudinal spin-echo images acquired in the absence and presence of pumping power. (b2) and (c2) contain one-dimensional SNR profiles (solid lines) and gradient profiles (dotted lines) plotted along the dashed line in (b1) and (c1). The green dashed lines in (b2) and (c2) correspond to twice the standard deviation evaluated over the entire gradient plot. Abbreviation: RCIA = Right Common Iliac Artery, LCIA = Left Common Iliac Artery, AA = Abdominal Aorta.

Figure 3a is the transverse image acquired immediately below the aortic bifurcation to locate the position of the endo-colon WAND. The dashed line passing through the left and the right common iliac arteries defines the orientation of longitudinal images to be acquired subsequently. Figure 3b1 and c1 are zoomed-in views of longitudinal images acquired without and with the pumping field. The passive resonator cannot clearly observe medial walls of common iliac arteries (Fig. 3b1), while the active resonator can image vascular walls with much improved quality (Fig. 3c1). Compared to the Signal-to-Noise-Ratio profile of the passive resonator (solid line) in Fig. 3b2, the active resonator has about five times the sensitivity of the passive resonator (Fig. 3c2) for most non-void regions in the plot. This enhanced sensitivity enables clear identification of luminal boundaries whose SNR gradients (dotted line) exceed twice the standard deviation (green dashed line) evaluated over the entire gradient plot. In comparison, the peak corresponding to the media boundary of left common iliac artery is missing in Fig. 3b2, due to the lower sensitivity of the passive resonator.

**Figure 4:**
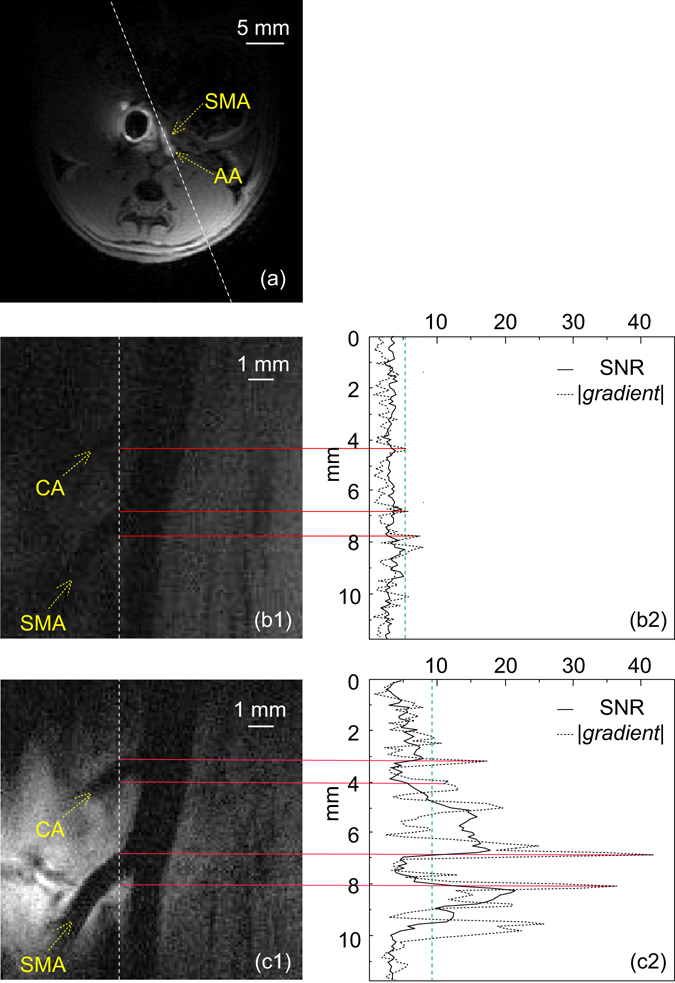
*In vivo* images of Superior Mesenteric Artery and Celiac Artery. (**a**) The transverse slice to locate the junction between the superior mesenteric artery and the abdominal aorta with the passive resonator inserted inside the rectal. (b1) and (c1) are zoomed-in views of the longitudinal spin-echo images acquired in the absence and presence of pumping power. (b2) and (c2) contain one-dimensional SNR profiles (solid lines) and gradient profiles (dotted lines) plotted along the dashed line in (b1) and (c1). The green dashed lines in (b2) and (c2) correspond to twice the standard deviation evaluated over the entire gradient plot. Abbreviation: CA = Celiac Artery, SMA = Superior Mesenteric Artery, AA = Abdominal Aorta.

In addition to vessels in close vicinity to the endo-rectal detector, the WAND can also maintain its sensitivity advantage for regions that are further away. As shown in Fig. 4a, the aorta’s junction with the superior mesenteric artery is about 4 mm away from the detector’s surface. This is a distance separation larger than the detector’s own diameter. But compared to the image obtained by the passive resonator (Fig. 4b1), the active detector can clearly “sharpen” the views of vascular boundaries (Fig. 4c1). The sensitivity enhancement effect of the active detector is also quantitatively illustrated by comparing SNR profiles (solid lines) in Fig. 4b2 and c2. As shown in Fig. 4c2, the boundaries for both the celiac artery and the superior mesenteric artery can be clearly identified by the corresponding peaks in the gradient plot (dotted line) that exceed twice the standard deviation (green dashed line). In comparison, the peak corresponding to the upper boundary of the celiac artery is missing in Fig. 4b2, when the resonator is in its passive state.

## Discussion

In this work, a Wireless Amplified NMR Detector (WAND) with a cylindrically symmetric detection profile is constructed and non-surgically inserted into the rodent rectal to sensitively observe deep-lying abdominal vessels. This detector is compactly integrated with a parametric amplifier that can efficiently harvest wirelessly provided pumping power for *in situ* signal amplification. These locally amplified MR signals are then wirelessly transmitted to a standard commercial coil placed outside the body, without the need for specialized hardware interface. Although parametric amplification is slightly noisier than transistor based amplification, the WAND retains ~72% the sensitivity of a directly connected detector of the same dimension, which is still far more sensitive than the external surface coil. Compared to previous versions of WANDs^8, 9, 11^, the detector described here has improved cylindrical symmetry without the need for orientation adjustment (Supplementary Fig. S1). The detector uses its end rings to create the higher frequency mode for wireless power harvesting, and its vertical legs to create the lower frequency mode for MR signal reception and wireless signal transmission. Owing to the geometric orthogonality between its higher and lower frequency modes, the WAND can utilize the longitudinally applied pumping field to amplify MR signals from the transverse plane. This geometric configuration is perfect for endo-rectal and endo-esophageal applications where the detector is aligned approximately parallel to the B_0_ field by terminal segments of the digestive track. In the future, when the WAND is used as a swallowable device that can travel tortuously through the entire digestive track, its wireless amplification capability will still remain effective for the majority of orientations, as long as the detector’s long axis is not perfectly perpendicular to the pumping field. Such a swallowable device could be used to sensitively observe multiple organs along the digestive track, including the pancreas that is too deep to image at high resolution with external detectors. The WAND may also be used to enhance the detection sensitivity of heteronuclear^25^ or spectroscopic imaging^26, 27^, providing biochemical information about abdominal organs.

## Conclusion

The detection sensitivity of abdominal vessels can be greatly improved with a cylindrically symmetric Wireless Amplified NMR Detector (WAND) that can approach the region of interest through non-surgical insertion into the lower digestive track. The detector is convenient to operate owing to its cylindrical symmetry. It can achieve up to 21-times sensitivity gain over the external receiver coil for immediately adjacent regions, and maintain at least 5-times gain even for distance separations larger than one diameter away from its cylindrical surface. Such detection capability enables high resolution imaging of vascular walls that are deep lying inside the abdomen. Compared to hard-wired connections that are often inconvenient or impractical for interventional or implantable detectors, wireless powered amplification provides an alternative route of MR signal transmission for improved operation flexibility^13, 28–30^.

## Electronic supplementary material

Supplementary Information
