# Supplementary material for: Wireless MRI Colonoscopy for Sensitive Imaging of Vascular Walls
Source: Sci Rep. 2017 Jun 26;7:4228. doi: 10.1038/s41598-017-03902-7 (PMC5484665; doi:10.1038/s41598-017-03902-7)
Supplement: Supplementary file 1 — Supplementary Information [file 41598_2017_3902_MOESM1_ESM.pdf]

Wireless MRI Colonoscopy for Sensitive Imaging of Vascular Walls

Xianchun Zeng[1,2,4,&], Liangliang Chen[3,4,&], Chuan Wang[3], Jian Wang[1*], Chunqi Qian[3,4*]

[1]Department of Radiology, Southwest Hospital, Third Military Medical University, Chongqing, China.

[2]Department of Radiology, Guizhou Provincial People's Hospital, Guiyang, China.

[3]Department of Electrical and Computer Engineering, Michigan State University, East Lansing, MI, USA.

[4]Department of Radiology, Michigan State University, East Lansing, MI, USA.

& These two authors contribute equally to this work.

Correspondence:

Jian Wang

30 Gaotanyan Rd, Chongqing, China, 400038

Tel: +86 (23) 68754419; Fax: +86 (23) 65463026

Email: wangjian_811@yahoo.com

Chunqi Qian

846 Service Rd, East Lansing, MI, 48824

Tel: +1 (517) 884-3292; Fax: +1 (517) 432-2849

Email: qianchu1@msu.edu

Improved detector designs for better symmetry and homogeneity

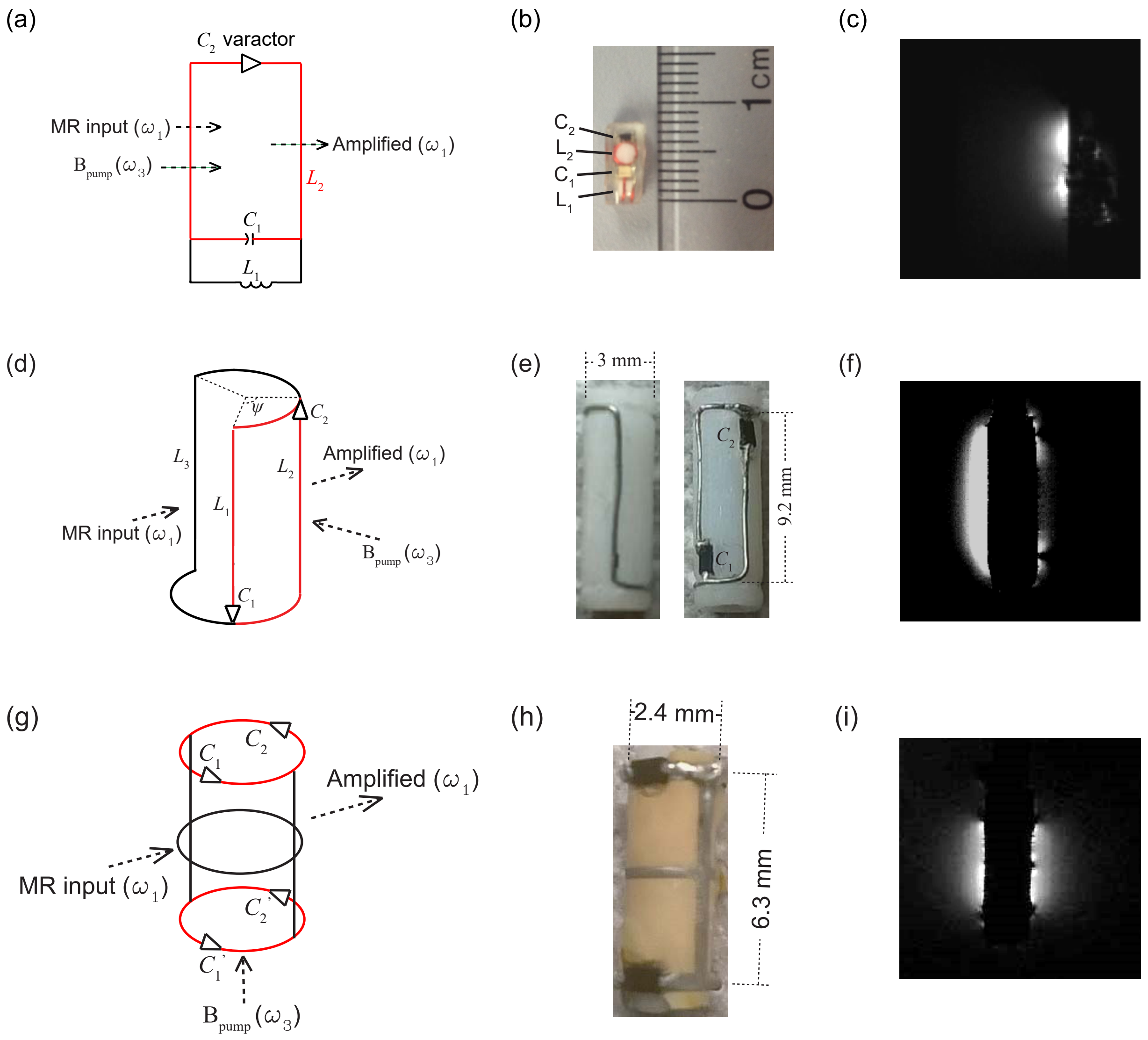

Supplementary Fig. 1

The schematic diagram (a) and the picture (b) of the original version of WAND[9] based on a double resonance Foster network. This detector has approximate left-right symmetry. Its constituting inductors L_1 (labelled in black) and L_2 (labelled in red) are separated by the chip capacitor C_1. As a result, the detector has inhomogeneous detection profile for the slice acquired along its symmetric plane (c).

The schematic diagram (d) and the pictures (e) of a recent version of WAND based on a cylindrical circuit[11]. In addition to left-right symmetry, this cylindrical detector has up-down symmetry with a homogeneous longitudinal detection profile. But according to the longitudinal image acquired through its symmetric plane (f), regions closer to the rectangular loop containing the two varactors (C_1 and C_2) has lower signal intensity. This is because the rectangular loop is designed to receive the pumping signal rather than MR signals.

The schematic diagram (g) and the picture (h) of a cylindrically symmetric WAND reported in this paper. As shown in (i), this detector has cylindrical symmetry with good longitudinal and azimuthal homogeneity.
